# Acute Pneumonia Caused by Clinically Isolated *Legionella pneumophila* Sg 1, ST 62: Host Responses and Pathologies in Mice

**DOI:** 10.3390/microorganisms10010179

**Published:** 2022-01-14

**Authors:** Jiří Trousil, Lucia Frgelecová, Pavla Kubíčková, Kristína Řeháková, Vladimír Drašar, Jana Matějková, Petr Štěpánek, Oto Pavliš

**Affiliations:** 1 Institute of Macromolecular Chemistry, Czech Academy of Sciences, Heyrovského nám. 2, 162 00 Prague, Czech Republic; stepanek@imc.cas.cz; 2 Department of Pathological Morphology and Parasitology, Faculty of Veterinary Medicine, University of Veterinary Sciences Brno, Palackého tř. 1946/1, 612 42 Brno, Czech Republic; frgelecoval@vfu.cz; 3 Military Health Institute, Military Medical Agency, Tychonova 1, 160 00 Prague, Czech Republic; pafcule@centrum.cz (P.K.); oto.pavlis@email.cz (O.P.); 4 Small Animal Clinical Laboratory, Faculty of Veterinary Medicine, University of Veterinary Sciences Brno, Palackého tř. 1946/1, 612 42 Brno, Czech Republic; rehakovak@vfu.cz; 5 National Legionella Reference Laboratory, Public Health Institute Ostrava, Masarykovo náměstí 16, 682 01 Vyškov, Czech Republic; Vladimir.Drasar@zuova.cz; 6 Department of Medical Microbiology, Second Faculty of Medicine, Charles University, Motol University Hospital, V Úvalu 84, 150 06 Prague, Czech Republic; jana.matejkova@fnmotol.cz

**Keywords:** legionellosis, histopathology, A/J mouse, lung infection, mouse model, inflammatory

## Abstract

Legionnaires’ disease is a severe form of lung infection caused by bacteria belonging to the genus *Legionella*. The disease severity depends on both host immunity and *L. pneumophila* virulence. The objective of this study was to describe the pathological spectrum of acute pneumonia caused by a virulent clinical isolate of *L. pneumophila* serogroup 1, sequence type 62. In A/JOlaHsd mice, we compared two infectious doses, namely, 10^4^ and 10^6^ CFU, and their impact on the mouse status, bacterial clearance, lung pathology, and blood count parameters was studied. Acute pneumonia resembling Legionnaires’ disease has been described in detail.

## 1. Introduction

Geographically, infectious diseases spread much faster now than at any time in history, and they continue to cause significant morbidity and mortality in human populations as well as economic disruption [1,2]. Taking bacterial infections as an example, humankind has been facing a crisis of antibiotic resistance because of the overuse and misuse of antibiotics. Bacterial pathogens can grow rapidly into biofilms that block the diffusion of antibiotics and thereby represent a public health problem. Some bacterial species, so-called intracellular bacteria, have acquired a complex and intricate ability to “hide” in mammalian cells, such as macrophages, thereby preventing antibiotics from destroying hidden pathogens and further increasing the challenges of therapy [3].

*Legionella pneumophila* is an emerging facultative intracellular bacterium that lives in both water and soil. It proliferates intracellularly, mostly in protozoa, including amoebae and ciliates. Once contaminated water droplets are inhaled by a human host, the bacteria reach the pulmonary alveoli, where they are phagocytized by resident alveolar macrophages. Depending on the host immunity and *L. pneumophila* virulence, the infection may progress to severe acute pneumonia, which is called Legionnaires’ disease, a milder illness called Pontiac fever, and possibly bacteremia resulting in extrapulmonary manifestations [4,5], including myocarditis, acute disseminated encephalomyelitis, and multiorgan failure [6,7,8,9]. *L. pneumophila* is a commonly detected bacterial pathogen in pneumonia-suffering patients admitted to intensive care units [10]. *Legionella* represents a common cause of community- and hospital-acquired pneumonia and occurs sporadically or in clusters and outbreaks [11].

The genus *Legionella* includes 62 described species, including *L. longbeachae*, *L. bozemanii*, *L. micdadei*, etc. [12,13]. However, only *L. pneumophila* is regarded as a significant pathogen, and it constitutes the majority of clinical isolates. Of note, *L. pneumophila* serogroup (Sg) 1 appears to be predominant [14,15]. Epidemiological studies have reported significant geographic variation in the seroprevalence of legionellosis both globally and domestically. In this regard, the combination of standardized sequencing methods with monoclonal antibody (MAb) typing has been described as a useful approach for both epidemiological investigations. Knowledge of a particular sequence type (ST) enables the assessment of a possible health risk. Monoclonal antibodies in the Dresden panel can be used to subdivide the emerging Sg 1 into 10 different subgroups. The Pontiac subgroup containing strains with the virulence-associated epitope MAb 3/1 prevails among clinical isolates of *L. pneumophila* [16]. The data reported for Germany [17], the United Kingdom [18], and the United States [19] indicate that the vast majority (65% to 100%) of *L. pneumophila* Sg 1 clinical isolates are associated with MAb 3/1. However, based on environmental isolates, only a minority belonged to the MAb 3/1-positive group.

In this work, we report on a highly virulent clinical isolate from a patient who presented with severe acute pneumonia and septic shock with the etiological agent identified as *L. pneumophila* serogroup (Sg) 1, ST 62, which is in the top six strains internationally that cause disease [15]. Although the investigation of clinical cases is important to reveal the environmental source and suggest remedial measures, investigations of ST 62 clinical cases often remain unresolved with no bacterial source identified [20]. To date, only a few *L. pneumophila* STs have been used for in vivo infections in rodents since this emerging pathogen was first identified in 1976. For instance, considering Sg 1, attention has been focused on *L. pneumophila* 130b [21,22,23,24], with ST 42 referenced by Underwood and coworkers [25], *L. pneumophila* Corby ST 51 [26], and the type strain (ATCC 33152) Philadelphia ST 36 [26]. In the case of other works, STs were not referred [27,28]. However, many questions concerning legionellosis pathogenesis and infection biology remain unclear.

In this study, we focused, for the first time, on *L. pneumophila* Sg 1, ST 62, MAb Knoxville, which was isolated from a lower respiratory tract aspirate of a 64-year-old man with severe acute pneumonia and septic shock. To contribute to the field of *Legionella* infections in rodents, clinically isolated *L. pneumophila* was used to infect A/JOlaHsd mice, the only murine strain permissive for legionellosis. Furthermore, we studied the effects on mouse pathology that resembled human pneumonia. To our knowledge, this is the first report on ST 62 infection in mice.

## 2. Material and Methods

### 2.1. Mice

Specific pathogen-free female A/JOlaHsd mice were obtained from Envigo RMS B.V., NM Horst, Netherlands. A/JOlaHsd females between the ages of 6 and 7 weeks were used for all the experiments, and they were housed in HEPA-filtered racks (Tecniplast S.p.A., Buguggiate VA, Italy) with a 12:12-h light-dark cycle. The diet used was Altromin 1324 F (Altromin GmbH & Co. KG, Lage, Germany). All animal studies were conducted according to Czech law No. 246/1992 Sb. on animal protection against brutalization and were approved by the Ethics Committee of the Ministry of Defence of the Czech Republic.

### 2.2. Bacteria Identification and Cultivation

The clinical isolate of *L. pneumophila* serogroup (Sg) 1, sequence type (ST) 62, MAb Knoxville was obtained from a male patient with severe pneumonia caused by *L. pneumophila*. The strain was identified using MALDI-TOF and serologically with the DrySpot™ Legionella Pneumophila Serogroup 1 Latex Agglutination Test (Oxoid, Hampshire, UK). Further typing was carried out using an international panel (also known as Dresden panel) of seven monoclonal antibodies [16,29]. Genotyping of the isolate was performed using the seven-gene protocol from the ESGLI SBT scheme [30]. For this, an online Sequence-Based Typing (SBT) Database tool was used to assign individual ST numbers. Based on the typing described, the isolate was identified as Sg 1, ST 62, MAb Knoxville. To prevent possible changes in strain virulence, the bacteria were stored with a minimum number of passages as glycerol stocks (tryptone soy broth supplemented with 30% glycerol) at –150 °C before the infection experiments.

The cultures were grown on buffered charcoal yeast extract (BCYE) agar in a humidified atmosphere at 37 °C for 3–5 days. The handling of *L. pneumophila* was carried out in accordance with the regulations of the State Office for Nuclear Safety of the Czech Republic.

### 2.3. Mice Infection

A/JOlaHsd mice were randomly divided into groups of 5 animals. They were then anesthetized with xylazine (8 mg/kg, i.p.) and ketamine (80 mg/kg, i.p.) and intranasally (i.n.) infected with either 1 × 10^4^ or 1 × 10^6^ CFU of *L. pneumophila* (50 μL per mouse, PBS). One hour post-infection (hpi), two mice were euthanized to control the initial number of bacteria in both the lungs and spleen. Subsequently, the CFU in the tissues was assessed at four different time points (3–10 days post-infection, dpi). The mice were euthanized, and dissected organs were homogenized in tea strainers using sterile saline. The resulting serially diluted homogenates were plated on glycine vancomycin polymyxin cycloheximide (GVPC) BCYE agar plates (Oxoid Ltd., Brno, Czech Republic) and incubated in a humidified atmosphere containing 5% CO_2_ at 37 °C for 3–5 days. Two mice from each group were taken for histopathological examination. For this, the organs were photodocumented and collected into specimens with a fixing solution (see below). Blood samples were taken from all the animals.

All animal challenges were carried out in a select agent-approved, restricted-access laboratory.

### 2.4. Hematological Analysis

At each time point of the infection experiment, terminal blood collection was carried out by axillary incision, with blood collected using a sterile EDTA-rinsed Pasteur pipette, placed into sterile tubes enriched with ethylenediaminetetraacetic acid tripotassium salt (0.5 mL Tapval Aquisel, Barcelona, Spain), and stored at 4 °C before the analysis. A Sysmex XT-2000iv analyzer (Sysmex, Sysmex America Inc., Lincolnshire, IL, USA) was used for routine hematological analysis; in the case of an abnormal white blood cell (WBC) scattergram, a microscopic WBC differential count from routinely prepared blood smears followed (Hemacolor, Merk, Germany).

### 2.5. Histopathological Examination

The organs of the mice (lung and spleen) were collected after euthanasia and prepared for histopathological examination. Tissues were fixed in buffered 10% neutral formalin, dehydrated, embedded in paraffin wax, and sectioned on a microtome at a thickness of 4 μm. Tissue sections from the organs of all mice were stained with hematoxylin and eosin (H&E). A Warthin–Starry (WS) silver staining kit (010270, Diapath S.p.A., Martinengo, Italy) was used for the proof of bacteria. Masson’s trichrome staining was performed to stain collagen connective tissue. In addition, immunohistochemistry was conducted on the samples to confirm the presence of macrophages (CD11b+). For this, rabbit monoclonal anti-CD11b antibody (1/4000 dilution, ab133357, Abcam, Cambridge, United Kingdom) and a Specific HRP/DAB IHC detection kit micropolymer (ab236466, Abcam) were used according to the manufacturer’s conditions.

### 2.6. Data Analysis

The results are expressed as the means ± SD. Data were analyzed by ANOVA and Tukey’s post hoc tests. Differences were considered significant at * *p* ≤ 0.05, ** *p* ≤ 0.01, and *** *p* ≤ 0.001.

## 3. Results

### 3.1. Case Report and Isolate Characterization

A 64-year-old man presented to the pulmonology clinic with exacerbated chronic obstructive pulmonary disease (COPD). On day 2, his respiratory status further deteriorated, and he developed multiorgan dysfunction and severe septic shock, requiring ICU admission. Notably, laboratory reports revealed C-reactive protein (CRP) at 340 mg/L, procalcitonin (PCT) at 65 µg/mL, and leukocytosis at 24 × 10^9^/L, suggesting a severe condition. The initial microbiological diagnostics were as follows: lower respiratory tract aspirate was assayed by PCR (FilmArray^®^ Pneumonia Panel) with negative results for viral respiratory tract infections. The only positive result of the diagnostics was obtained for *L. pneumophila*, and the urinal antigen for *L. pneumophila* was also positive. Ciprofloxacin (400 mg i.v. every 12 h) and rifampicin (450 mg i.v. every 12 h) were initiated as treatment. On day 3, the patient’s condition was critical, and he was placed on veno-arterial extracorporeal membrane oxygenation (ECMO). Vasopressor support and coagulopathy correction were initiated. Both lower limbs began to reveal signs of ischemia the next day, which was presumably due to vasopressor support and septic shock.

On day 5, antibiotic treatment was combined with ampicillin/sulbactam (3 g i.v. every 8 h). On day 9, because a prolonged course of ventilator care was expected, a tracheostomy was performed. Hemodynamics and oxygenation parameters began to stabilize; thus, ECMO support was weaned. However, the lower respiratory tract aspirate revealed candidiasis. Notably, during ICU admission, *C. albicans*, *C. glabrata*, and *C. fabiani* were found alternatively, and fluconazole was used (400 mg i.v. every 24 h). ECMO was decannulated on day 10. Over the following days, an air leak occurred, and the patient suffered from subcutaneous emphysema as a complication of a chest drain; thus, redrainage and CT controls were needed. Furthermore, inflammatory parameters slowly began to resolve. On day 12 of admission, a control laboratory report revealed CRP of 171 mg/L, PCT of 8.5 µg/mL, and leukocyte count of 16 × 10^9^/L. The *Candida* sp. episode continued.

On day 23, laboratory reports revealed CRP of 26 mg/L, PCT of 0.35 µg/mL, and leukocytes of 6 × 10^9^/L, and *Sphingomonas paucimobilis* was found in lower respiratory tract aspirate. The antimicrobial regimen consisted of ciprofloxacin (400 mg i.v. every 8 h), azithromycin (500 mg i.v. every 24 h), and fluconazole (400 mg i.v. every 24 h). Over the next days, both respiratory tract aspirates and central venous catheters were positive for *Pseudomonas aeruginosa*, with a simultaneous increase in inflammatory parameters. Notably, the patient later began to develop toe necrosis as a result of peripheral ischemia. The patient underwent necrotizing soft tissue debridement and transmetatarsal amputation of the right foot toes under general anesthesia on day 31. On day 40, right transtibial amputation under general anesthesia was necessary due to necrosis and ischemia. Over the next admission, repeated microbiology diagnostics revealed *P. aeruginosa* altered by *Elisabethkingia meningoseptica* and *L. pneumophila* in the lower respiratory tract aspirate, resulting in a complex antibiotic intervention (supplemented with antifungals) that was finally successful. The patient was discharged after 69 days from admission based on antibiotic therapy. He was followed up at the rehabilitation clinic of the same hospital.

The etiological agent of the initial pneumonia episode was identified by both MALDI-TOF and PCR as *L. pneumophila* and further typed as Sg 1, ST 62, MAb Knoxville. The clinical isolate was further used for A/JOlaHsd mouse (see below) infection and tackling the resulting pathology.

### 3.2. Body Weight and Bacterial Burden

We intranasally (i.n.) infected A/JOlaHsd females with an *L. pneumophila* suspension containing either 10^4^ CFU, which was referred to as the low dose, and 10^6^ CFU, which was referred to as the high dose. The infection was confirmed by the initial, estimated number of bacteria delivered to the lungs at time 0 (0 days post-infection, dpi). The presence of *L. pneumophila* bacilli was also noted by histochemistry (see below). Over the ensuing 10 days, the mice were observed and assessed for subacute general appearance, bacterial load in both lungs and spleen, blood count, and histopathology.

**Figure 1:**
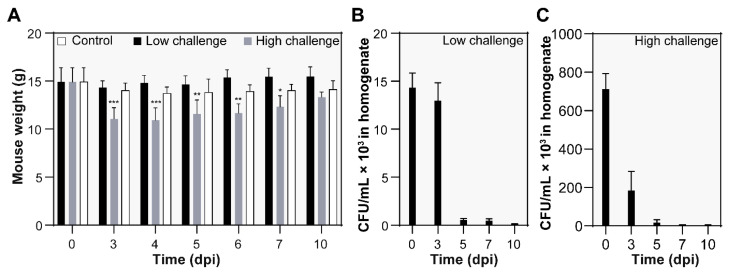
General appearance and clinical signs of intranasally A/JOlaHsd mice inoculated with the *L. pneumophila* isolate. (**A**) Body weight course during the infection. Statistical significance was evaluated compared to the control group by ANOVA. (**B**,**C**) *L. pneumophila* Sg 1, ST 62, Mab Knoxville burden in lungs. At specific time points of both the low (**B**) and high dose (**C**) challenge, the animals were sacrificed, and the CFU value was estimated in lung tissue homogenates. The results indicate mean values ± SD. Asterisks indicate statistical significance compared with the noninfected control (* *p* ≤ 0.05, ** *p* ≤ 0.01, and *** *p* ≤ 0.001).

Reduced activity, lethargy, and ruffled fur were observed during the ensuing ca. 1–4 dpi. As depicted in Figure 1A, transient weight loss occurred, and it was notable and significant only in the case of the high dose of the *L. pneumophila* Sg 1, ST 62, MAb Knoxville clinical isolate. One mouse inoculated with the high dose died at 5 dpi and was further examined by histopathology (see below).

In the case of both the low and high *L. pneumophila* challenge, the bacterial burden was confirmed by CFU estimation (Figure 1B,C), followed by bacterial clearance at 10 dpi. Additionally, we determined whether the infected mice suffered from disseminated infection. To this end, the CFU was estimated in spleen homogenates in the case of both the low- and high-dose-challenged mice. Notably, *L. pneumophila* bacilli were recovered from spleens from mice inoculated with a high dose, which indicates hematogenic/lymphogenic dissemination. We were not able to culture disseminated bacteria from spleen tissues from the low-dose-challenged mice.

### 3.3. Hematological Analysis

**Figure 2:**
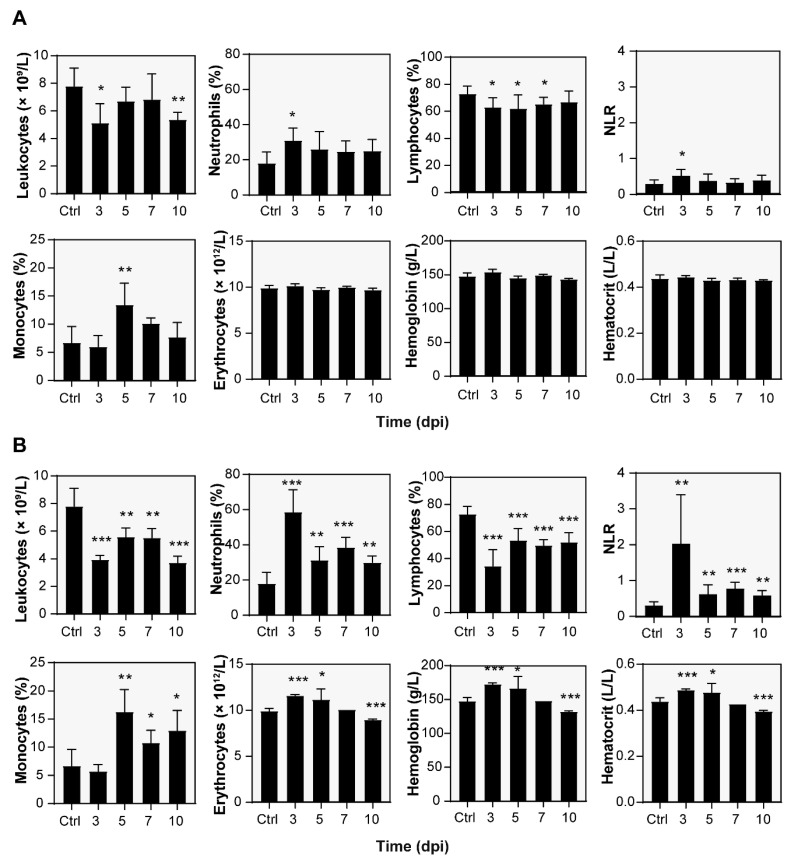
Selected parameters of the hematological analysis. Mice inoculated either with a low (**A**) or high (**B**) dose of *L. pneumophila* Sg 1, ST 62, or MAb Knoxville were anesthetized, and terminal blood collection was carried out. Data represent the mean ± SD; asterisks indicate statistical significance ((* *p* ≤ 0.05, ** *p* ≤ 0.01, and *** *p* ≤ 0.001) compared with the control (Ctrl, noninfected animals). The neutrophil-to-lymphocyte ratio is abbreviated as NLR.

At specific time points of the infection challenges, the blood of A/JOlaHsd mice was examined for blood counts and white blood cell differential over the ensuing 10 days (Figure 2). White blood cell counts suggested a severe acute infection. As a result of pneumonia induction, both infection doses led to a decrease in leukocyte count (Figure 2A,B). Low- and high-infection dose-challenged mice had leukocyte counts of 5.1 ± 1.4 × 10^9^/L (*p* < 0.05) and 3.9 ± 0.3 × 10^9^/L (*p* < 0.001), respectively, at 3 dpi. Note that the control leukocyte count was 7.8 ± 1.3 × 10^9^/L.

The leukocyte count decrease was caused by the reduction in lymphocytes from 73 ± 6% (5.6 ± 1.0 × 10^9^/L) estimated for the control mice to 63 ± 7% (4.3 ± 0.3 × 10^9^/L, *p* < 0.05) and 35 ± 12% (1.4 ± 0.5 × 10^9^/L, *p* < 0.001) in the case of the low and high doses, respectively. At the same time point, the relative neutrophil counts were significantly increased in the case of both the low (31 ± 7%, *p* < 0.05) and high (59 ± 13%, *p* < 0.001) infection doses, which corresponded to absolute counts of 1.6 ± 0.7 × 10^9^/L and 2.3 ± 0.3 × 10^9^/L, respectively. The control count was 18 ± 6% or 1.4 ± 0.63 × 10^9^/L as the absolute count.

The monocyte cell counts were also significantly increased; however, the maximum counts shifted to 5 dpi. However, the control monocyte counts were 7 ± 3% (0.5 ± 0.3 × 10^9^/L as the absolute count), the high and low doses led to 14 ± 4% (0.8 ± 0.1× 10^9^/L) and 16.3 ± 4.0% (0.8 ± 0.3 × 10^9^/L) at 5 dpi, respectively. Both changes were statistically significant (*p* < 0.01) compared to the control mice.

When the erythrocyte counts were examined, there was a notable increase (*p* < 0.001) in the high-dose-challenged mice (10.2 ± 0.2 × 10^12^/L) compared to the control mice (9.9 ± 0.3 × 10^12^/L) at 3 dpi. This red blood cell count change was transient and, compared to the noninfected control, further led to a statistically significant (*p* < 0.001) decrease in erythrocyte count values at 10 dpi. The total blood hemoglobin and hematocrit changes followed the same time-dependent manner. A low infection dose did not affect the red blood cell parameters discussed.

Note that the mean corpuscular volume (MCV), mean cell hemoglobin (MCH), mean corpuscular hemoglobin concentration (MCHC), and red blood cell distribution width (RDW) was not changed significantly relative to the noninfected control (Figure S1) in either infection challenge.

### 3.4. Gross Pathology Appearance and Histopathological Changes

**Figure 3:**
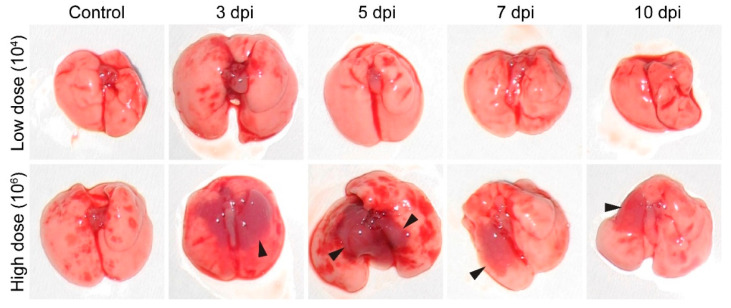
Gross pathology of the lungs in A/JOlaHsd mice. The animals were sacrificed by terminal blood collection under anesthesia, and the gross pathology of the lungs was observed. Photographs of gross lung specimens collected at 3, 5, 7, and 10 dpi are shown. Note the dark red areas in the lung parenchyma (black arrowheads). Tissue from noninfected, healthy animals is referred to as a control.

The pathological changes in the lungs of both the low- and high-dose-challenged mice were examined during infection. No significant pathology was observed in the case of mice infected with the low dose compared to the healthy control (Figure 3). In contrast, in the case of the high infection dose, within 3 to 10 dpi, localized large, edematous, and dark red areas were notable on the lung surface and parenchyma (Figure 3, black arrowheads), representing severe lung condition and areas of consolidation.

**Figure 4:**
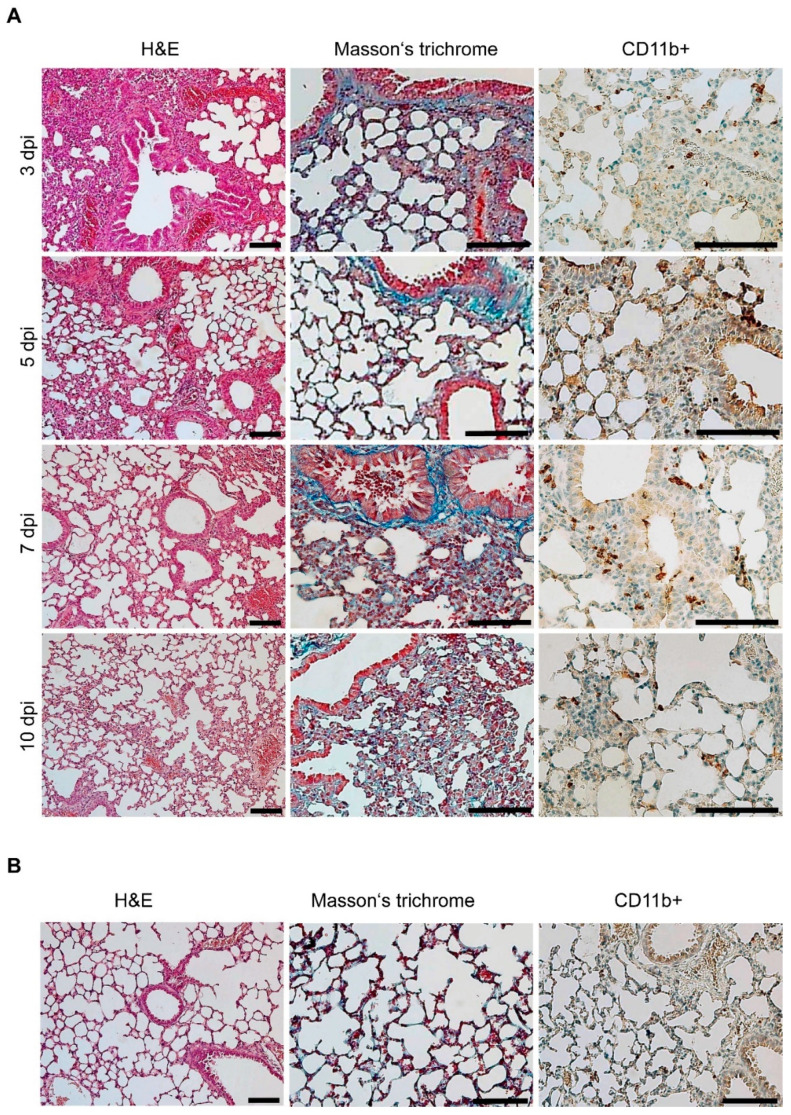
Lung tissue histopathological appearance of a mouse infected with a low dose of the clinical isolate. (**A**) Hematoxylin and eosin (H&E), Masson’s trichrome, and macrophage (CD11b+, brown) staining was performed on the lung tissue sections from a representative A/JOlaHsd mouse infected with the *L. pneumophila* Sg 1, ST 62, MAb Knoxville clinical isolate at a dose of 10^4^ CFU i.n. Note that the blue structures (Masson’s trichrome) were referred to as an outcome of normal connective tissue septa compression due to the inflammatory reaction. (**B**) Lung sections obtained from noninfected A/JOlaHsd mice (noninfected control). Scale bars: 100 µm.

Histopathological examination of lung samples from both infectious challenges demonstrated similar results but with different intensities and severities. Low-dose challenge-related histopathological findings are depicted in Figure 4A, and uninfected control histopathology is depicted in Figure 4B.

An interstitial and peribronchial reaction characterized by multifocal to coalescing infiltration of neutrophils, lymphocytes, plasma cells, and macrophages was present within 3 and 5 dpi. Part of the lung parenchyma remained normal, and the inflammatory reaction was mostly localized without a significant trend toward peripheral expansion. Infiltration of tissue with neutrophils began to decrease or was observed occasionally within 5 and 10 dpi, which coincided with the increased neutrophil blood counts discussed above (cf. Figure 2). CD11b+ cells with morphological characteristics of macrophages were demonstrated by immunohistochemistry within all the lung tissue sections examined. Pulmonary interstitial hyperemia was observed within 3 and 10 dpi. Masson’s trichrome did not reveal fibrosis in the sites of inflammation during the low-dose challenge.

**Figure 5:**
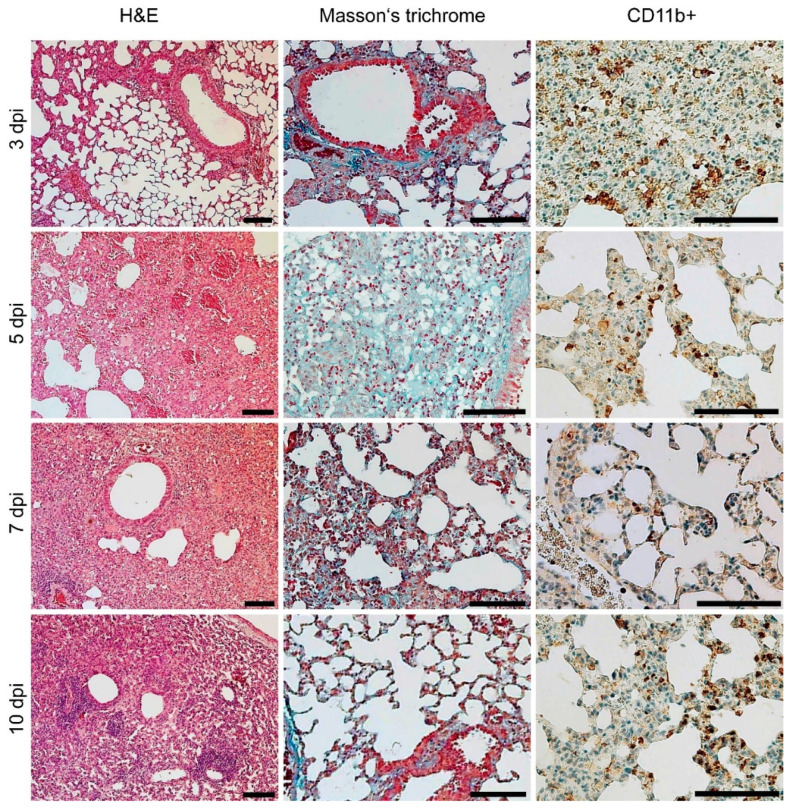
Histopathological appearance of high-dose-infected murine lungs. Hematoxylin and eosin (H&E), Masson’s trichrome, and macrophage (CD11b+, brown) staining was performed on the lung tissue sections from a representative A/JOlaHsd mouse infected with the *L. pneumophila* Sg 1, ST 62, MAb Knoxville clinical isolate at a dose of 10^6^ CFU i.n. Note that the blue structures (Masson’s trichrome) were referred to as an outcome of normal connective tissue septa compression due to the inflammatory reaction. Scale bars: 100 µm.

**Figure 6:**
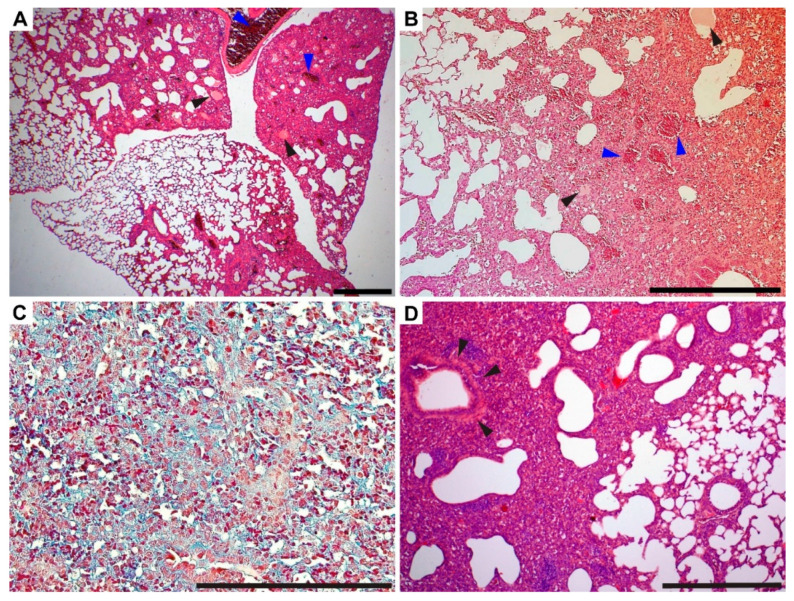
Heterogeneity of *L. pneumophila* Sg 1, ST 62, Mab Knoxville acute infection. Histopathological lung sections demonstrate multiple pathological changes, such as hyperemia (**A**, blue arrowheads), alveolar edema (**A**,**B**, black arrowheads), hemorrhage (**B**, blue arrowheads), fibrosis (**C**, blue staining), and atelectasis with severe diffuse inflammation (**D**, black arrowheads). The sections are related to high-dose-challenged mice euthanized at 5 dpi (**A**,**B**) and 10 dpi (**C**,**D**). H&E (**A**,**B**,**D**) and Masson’s trichrome (**C**) are shown; scale bars: 500 µm.

The high-dose challenge of the A/JOlaHsd mice revealed more severe pathological changes (Figure 5). The high legionellae dose resulted in a multifocal to coalescing reaction characterized by mixed inflammatory infiltration at 3 dpi. At 5 dpi, the lung condition progressed to diffuse and coalescing inflammation, which was notably more severe than the low-dose challenge. Hyperemia (Figure 6A, blue arrowhead) and foci of alveolar edema (Figure 6B, black arrowheads) were present as well as areas of extensive or diffuse hemorrhages (Figure 6B, blue arrowheads) within the lung parenchyma, which was in line with the gross lung appearance (cf. Figure 4). Hemorrhage was limited to the areas of inflammation. The numbers of infiltrating neutrophils were decreased. Mononuclear diffuse inflammatory reaction, together with hyperemia, was present within 5 and 10 dpi, and macrophage presence was proven by CD11b+ cell staining. Neutrophilic infiltration was rarely observed and mostly appeared as rare foci. Atelectasis (Figure 6D), which resulted from the compression of alveoli together with the beginning of fibrosis (Figure 6C), was present at 10 dpi.

**Figure 7:**
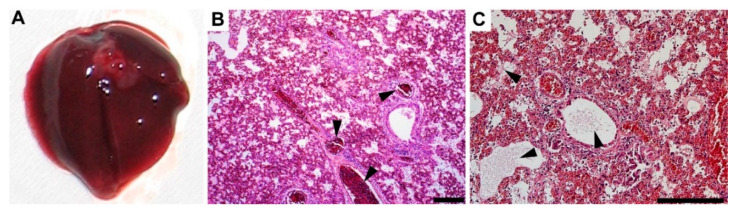
Postmortem examination (5 dpi) of a dead mouse infected with a high dose of the clinical isolate. (**A**) Gross pathology of lungs dissected from the dead mouse. Note the diffuse dark red coloration of parenchyma. (**B**) Condition showing hyperemia of the parenchyma with dilated vessels filled with blood (black arrowheads). (**C**) Diffuse alveolar damage with intra-alveolar fibrin deposits (black arrowheads). H&E staining, scale bars: 200 µm.

**Figure 8:**
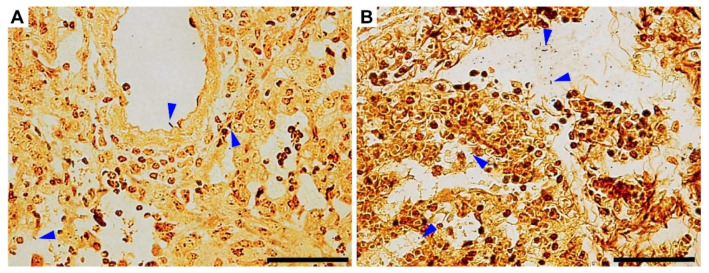
Bacterial visualization within the lungs of specific pathogen-free A/JOlaHsd mice infected with a high *L. pneumophila* dose. Rod-shaped structures, presumably *L. pneumophila,* were revealed (blue arrowheads). (**A**,**B**) Warthin–Starry silver stain for bacterial cells, appearing dark brown to black, as obtained at 5 dpi. (**B**) Postmortem examination (5 dpi) of a dead mouse; note the fibrin fibers within the lung parenchyma. Scale bars: 50 µm.

As mentioned above, one mouse infected with 10^6^ CFU i.n. died at 5 dpi, which was presumably due to severe pneumonia. Upon further examination, the dissected lungs showed diffuse dark red coloration with notable signs of a severe condition (Figure 7A, cf. Figure 3). H&E staining showed severe fibrinous bronchopneumonia, which was localized almost within the whole lung parenchyma. Mononuclear and neutrophilic infiltrates were noted in alveoli, bronchi, and interstitially. Both hyperemia (Figure 7B) and fibrin (Figure 7C and Figure 8B) were accentuated. Fibrosis was not observed.

Note that the histochemistry also revealed bacteria in all the lung sections, which was in line with the detection of *L. pneumophila* in the lung homogenates (CFU estimation). Localized mostly in alveoli, rod-shaped bacteria were noted using the Warthin–Starry silver staining method (Figure 8, blue arrowheads).

## 4. Discussion

The framework of this study was based on the case report presented above, as well as the emergence of legionellosis and intracellular infections. The etiological agent discussed was identified as *L. pneumophila* and further typed as Sg 1, ST 62. This ST is internationally in the top six *L. pneumophila* strains causing disease [15] and dominates among Czech clinical isolates of *L. pneumophila*, accounting for as much as 35% of all disease instances (unpublished data). Furthermore, the Dresden monoclonal antibody panel revealed MAb 3/1 positivity (the MAb Knoxville subtype), which is associated with higher virulence [31].

As outlined above, *L. pneumophila* infection has already been described in rodents. The well-known experimental infection described by Brieland and colleagues [21] was based on *L. pneumophila* Sg 1, ST 42. Note that there are no publications addressing infections caused by ST 62 in laboratory rodents. Herein, we, therefore, examined for the first time serial pathological changes in A/JOlaHsd mice suffering from pneumonia induced by the virulent clinical isolate ST 62. Complementary to Brieland’s report, we focused on the study of blood count and detailed histopathological examination. A/JOlaHsd mice were used since this strain is known as the only mouse strain revealing replicative *L. pneumophila* infection, which is due to genetic polymorphisms in the locus *lgn1*, which is within the *birc1e*/*naip5* gene that encodes a Nod-like receptor family member, rendering mice of the A/J background permissive to pulmonary replication of *L. pneumophila* [32].

As our results show, the intranasal challenge with *L. pneumophila* Sg 1, ST 62, and MAb Knoxville led to pneumonia, suggesting high virulence. In general, the mucosal epithelium of the respiratory tract serves as a barrier against systemic bacterial infections. Numerous pathogenic bacteria, however, possess virulence strategies that can overcome barriers and allow for dissemination to peripheral tissues [33]. Although rare, *L. pneumophila* has been reported to cause disseminated extrapulmonary infections, such as endocarditis or soft tissue infections [5,6,8,9]. Accordingly, we observed hematogenic/lymphogenic dissemination in the case of the high infection dose and reactive hyperplasia in the spleen, which was noted within follicle germinal centers (Figure S2A). The spleen disseminated burden was proven by both cultivation and the presence of bacteria, presumably legionellae, and visualization by the Warthin–Starry staining method (Figure S2B, blue arrowheads), which is in line with *L. pneumophila* Sg 1, ST 42 infection [21]. However, the number of bacteria found in spleen homogenates was relatively low. We found ca. 50–100 CFU per mL of spleen homogenate by 5 dpi, albeit no bacilli were detected from mice sacrificed on 7 and 10 dpi, which may indicate that the dissemination observed is only transient.

After severe pneumonia, a complete blood count and white blood cell differential count revealed the characteristics of an acute infection. At 3 dpi, it was characterized by a significant increase in the counts of neutrophils, which are the first responders to an infection and are rapidly mobilized into the blood to migrate to infected tissue [34]. This change was the most notable and consistent with neutrophil infiltration found by histopathological examination. Moreover, lymphocytes, which are an important group of white blood cells involved in both innate and adaptive immunity, were found to decrease secondary to the clinical status. In this context, the relationship between neutrophils and lymphocytes, also known as the neutrophil-to-lymphocyte ratio (NLR), was assessed. Compared to the noninfected control (Figure 2A,B), the NLR parameter was elevated significantly at 3 dpi, which was observed for both the low (*p* < 0.05) and high (*p* < 0.01) dose-infected mice. Significantly increased NLR values were observed by 10 dpi in the case of a high infection dose. The NLR has been suggested to be a useful marker to measure subclinical inflammation in humans, and it has been proven to correlate well with established inflammatory markers, such as the CRP, and has prognostic value in patients with malignancies [35,36]. In laboratory rodents, elevated NLR can reflect chronic stress [37,38], muscle damage [39], and staphylococcal mastitis [40]. Therefore, the elevated NLR parameter values appear to be consistent with the general appearance of and high dose-infected mouse status.

Similarly, the high-dose-challenged mice revealed an increased erythrocyte count and hemoglobin and hematocrit contents. Concerning the animal status presented above, we thus attribute these findings to a severe condition represented by lung edema and severe infection. The red blood cell parameters presumably reflect hemoconcentration secondary to increased capillary permeability and fluid loss [41,42]. This phenomenon was transient; however, a significant decrease in the red blood cell parameters was observed at 10 dpi, which likely occurred secondary to an inflammatory reaction [42].

Not surprisingly, severe lung pneumonia has been shown to affect gross lung appearance. Similarly, in our study, the high-dose-challenged mice revealed parenchyma-localized edematous areas. Note that similar appearances have been described in other murine lung infections, such as *Staphylococcus aureus* [43], *Stenotrophomonas maltophilia* [44], or influenza virus [45,46], thus indicating that the high dose challenge led to a severe acute condition of the A/JOlaHsd mice [47].

The clinical isolate *L. pneumophila* Sg 1, ST 62, MAb Knoxville infection led to a complex and dynamic orchestra of histopathological findings. The observed dynamics of histopathological changes in inflammation characteristics, lung condition severity, and decrease in neutrophil infiltration are in line with the blood count parameter and bacterial clearance time course, which can implicate parenchyma restoration dynamics. One may also hypothesize that neutrophil migration to *L. pneumophila*-infected lungs coincided with the development of substantial leukopenia, which suggests that both phenomena may be related, as also discussed by Sordi and coworkers [48]. Within the subacute time course, we did not observe significant signs of chronic inflammation and fibrosis. Only at the last time point (10 dpi, high-dose challenge) did fibrosis develop within the inflammatory foci (Figure 7C). Although this issue was not addressed further and remains unanswered, one could suggest that despite the somewhat rapid bacterial clearance (cf. Figure 1B,C) and the robust inflammatory response observed, the severe lung condition could lead to fibrosis-characterized healing that has been described in human *L. pneumophila* pneumonia [49].

Notably, the gross pathology of human acute pneumonia caused by *L. pneumophila* has revealed lobar or patchy lesions, consolidation, congestion, edema or focal hemorrhage (cf. Figure 4), as reviewed by Carrington [50]. Despite some semantic differences, human Legionnaires’ disease has been described as bronchopneumonia and diffuse alveolar damage, fibrin deposition, interstitial infiltration by neutrophils and macrophages/mononuclear cells, alveolar edema, and fibrosis [49,50]. This suggests that our experimental infection resembles the human disease.

Concerning the field of *L. pneumophila* pathogenesis and studies describing legionellosis in A/J mice, our study offers complementary information.

## 5. Conclusions

*L. pneumophila* Sg 1, ST 62, MAb Knoxville is a highly virulent sequencing type causing a severe infection in susceptible human hosts, as suggested by the case report described. The clinical isolate was characterized and further used for experimental infection in vivo and confirmed to be a highly virulent pathogen in A/JOlaHsd mice. Within 3 and 5 dpi, *L. pneumophila* Sg 1, ST 62, MAb Knoxville caused severe bronchopneumonia and presented diffuse inflammation, which was characterized by both mononuclear and neutrophil infiltration, hemorrhage, hyperemia, and alveolar edema. These pathologies, which were revealed in an infection dose-dependent manner, were supported by mouse appearance and blood parameter examination. Although A/JOlaHsd mice are not natural hosts of *Legionella* species, the induced experimental pneumonia resembled human Legionnaires’ disease. The results obtained contribute to the field of legionellosis.

## Data Availability

The authors confirm that the data supporting the findings of this study are available within the article and its supplementary materials.

## Supplementary Materials

The following supporting information can be downloaded at: https://www.mdpi.com/article/10.3390/microorganisms10010179/s1, Figure S1 Hematological analysis parameters. Figure S2 Disseminated *L. pneumophila* infection.

## Abbreviations

H&E: hematoxylin and eosin; MAb, monoclonal antibody; MCH, mean cell hemoglobin; MCHC, mean corpuscular hemoglobin concentration; MCV, mean corpuscular volume; NLR, neutrophil-to-lymphocyte ratio, RDW, red blood cell distribution width; Sg, serogroup; ST, sequence type.

## References

[1] Watkins K. (2018). Emerging Infectious Diseases: A Review. Curr. Emerg. Hosp. Med. Rep..

[2] Ellwanger J.H., Kaminski V.d.L., Chies J.A.B. (2019). Emerging infectious disease prevention: Where should we invest our resources and efforts?. J. Infect. Public Health.

[3] McClure E.E., Chávez A.S.O., Shaw D.K., Carlyon J.A., Ganta R.R., Noh S.M., Wood D.O., Bavoil P.M., Brayton K.A., Martinez J.J. (2017). Engineering of obligate intracellular bacteria: Progress, challenges and paradigms. Nat. Rev. Microbiol..

[4] Graham F.F., Hales S., White P.S., Baker M.G. (2020). Review Global seroprevalence of legionellosis - a systematic review and meta-analysis. Sci. Rep..

[5] Chitasombat M.N., Ratchatanawin N., Visessiri Y. (2018). Disseminated extrapulmonary Legionella pneumophila infection presenting with panniculitis: Sase report and literature review. BMC Infect. Dis..

[6] Burke P.T., Shah R., Thabolingam R., Saba S. (2009). Suspected Legionella-induced perimyocarditis in an adult in the absence of pneumonia: A rare clinical entity. Tex. Heart Inst. J..

[7] Bodur H., Savran Y., Koca U., Kilinç O., Albayrak S., Itil O., Akoglu S. (2006). Legionella pneumonia with acute respiratory distress syndrome, myocarditis and septic shock successfully treated with Drotrecogin Alpha (activated). Eur. J. Anaesthesiol..

[8] Sommer J.B., Erbguth F.J., Neundörfer B. (2000). Acute disseminated encephalomyelitis following Legionella pneumophila infection. Eur. Neurol..

[9] Ishimaru N., Suzuki H., Tokuda Y., Takano T. (2012). Severe Legionnaires’ disease with pneumonia and biopsy-confirmed myocarditis most likely caused by Legionella pneumophila serogroup 6. Intern. Med..

[10] Dietert K., Gutbier B., Wienhold S.M., Reppe K., Jiang X., Yao L., Chaput C., Naujoks J., Brack M., Kupke A. (2017). Spectrum of pathogen- and model-specific histopathologies in mouse models of acute pneumonia. PLoS ONE.

[11] Sabrià M., Campins M. (2003). Legionnaires’ Disease. Am. J. Respir. Med..

[12] Li L.-H., Zhang L., Wu H.-Y., Qu P.-H., Chen J.-C., Zhan X.-Y., Zhu Q.-Y., Chen C., Hu C.-H. (2021). Legionella septentrionalis sp. nov., isolated from aquatic environments in the northern PR China. Int. J. Syst. Evol. Microbiol..

[13] Yu V.L., Plouffe J.F., Pastoris M.C., Stout J.E., Schousboe M., Widmer A., Summersgill J., File T., Heath C.M., Paterson D.L. (2002). Distribution of Legionella Species and Serogroups Isolated by Culture in Patients with Sporadic Community-Acquired Legionellosis: An International Collaborative Survey. J. Infect. Dis..

[14] Jaber L., Amro M., Tair H.A., Bahader S.A., Alalam H., Butmeh S., Hilal D.A., Brettar I., Höfle M.G., Bitar D.M. (2018). Comparison of in situ sequence type analysis of Legionella pneumophila in respiratory tract secretions and environmental samples of a hospital in East Jerusalem. Epidemiol. Infect..

[15] Underwood A.P., Jones G., Mentasti M., Fry N.K., Harrison T.G. (2013). Comparison of the Legionella pneumophilapopulation structure as determined by sequence-based typing and whole genome sequencing. BMC Microbiol..

[16] Helbig J.H., Bernander S., Castellani Pastoris M., Etienne J., Gaia V., Lauwers S., Lindsay D., Lück P.C., Marques T., Mentula S. (2002). Pan-European study on culture-proven Legionnaires’ disease: Distribution of Legionella pneumophila serogroups and monoclonal subgroups. Eur. J. Clin. Microbiol. Infect. Dis..

[17] Reichardt K., Jacobs E., Röske I., Helbig J.H. (2010). Legionella pneumophila carrying the virulence-associated lipopolysaccharide epitope possesses two functionally different LPS components. Microbiology (Read. Engl.).

[18] Harrison T.G., Afshar B., Doshi N., Fry N.K., Lee J.V. (2009). Distribution of Legionella pneumophila serogroups, monoclonal antibody subgroups and DNA sequence types in recent clinical and environmental isolates from England and Wales (2000-2008). Eur. J. Clin. Microbiol. Infect. Dis..

[19] Kozak N.A., Benson R.F., Brown E., Alexander N.T., Taylor T.H., Shelton B.G., Fields B.S. (2009). Distribution of lag-1 alleles and sequence-based types among Legionella pneumophila serogroup 1 clinical and environmental isolates in the United States. J. Clin. Microbiol..

[20] David S., Mentasti M., Tewolde R., Aslett M., Harris S.R., Afshar B., Underwood A., Fry N.K., Parkhill J., Harrison T.G. (2016). Evaluation of an Optimal Epidemiological Typing Scheme for Legionella pneumophila with Whole-Genome Sequence Data Using Validation Guidelines. J. Clin. Microbiol..

[21] Brieland J., Freeman P., Kunkel R., Chrisp C., Hurley M., Fantone J., Engleberg C. (1994). Replicative Legionella pneumophila lung infection in intratracheally inoculated A/J mice. A murine model of human Legionnaires’ disease. Am. J. Pathol..

[22] Brieland J., Remick D., Freeman P., Hurley M., Fantone J., Engleberg C. (1995). In vivo regulation of replicative Legionella pneumophila lung infection by endogenous tumor necrosis factor alpha and nitric oxide. Infect. Imunn..

[23] Brieland J.K., Loebenberg D., Menzel F., Hare R.S. (2000). Efficacy of SCH27899 in an Animal Model of Legionnaires’; Disease Using Immunocompromised A/J Mice. Antimicrob. Agents Chemother..

[24] Edelstein P.H., Calarco K., Yasui V.K. (1984). Antimicrobial therapy of experimentally induced Legionnaires’ disease in guinea pigs. Am. Rev. Respir. Dis..

[25] Schroeder G.N., Petty N.K., Mousnier A., Harding C.R., Vogrin A.J., Wee B., Fry N.K., Harrison T.G., Newton H.J., Thomson N.R. (2010). Legionella pneumophila strain 130b possesses a unique combination of type IV secretion systems and novel Dot/Icm secretion system effector proteins. J. Bacteriol..

[26] Hawn T.R., Berrington W.R., Smith I.A., Uematsu S., Akira S., Aderem A., Smith K.D., Skerrett S.J. (2007). Altered inflammatory responses in TLR5-deficient mice infected with Legionella pneumophila. J. Immunol..

[27] Edelstein P.H., Edelstein M.A. (1999). In vitro activity of the ketolide HMR 3647 (RU 6647) for Legionella spp., its pharmacokinetics in guinea pigs, and use of the drug to treat guinea pigs with Legionella pneumophila pneumonia. Antimicrob. Agents. Chemother..

[28] Davis G.S., Winn W.C., Gump D.W., Craighead J.E., Beaty H.N. (1982). Legionnaires’ pneumonia after aerosol exposure in guinea pigs and rats. Am. Rev. Respir. Dis..

[29] Helbig J.H., Kurtz J.B., Pastoris M.C., Pelaz C., Lück P.C. (1997). Antigenic lipopolysaccharide components of Legionella pneumophila recognized by monoclonal antibodies: Possibilities and limitations for division of the species into serogroups. J. Clin. Microbiol..

[30] Qin T., Zhou H., Ren H., Guan H., Li M., Zhu B., Shao Z. (2014). Distribution of sequence-based types of legionella pneumophila serogroup 1 strains isolated from cooling towers, hot springs, and potable water systems in China. Appl. Environ. Microbiol..

[31] Fontana S., Scaturro M., Rota M.C., Caporali M.G., Ricci M.L. (2014). Molecular typing of Legionella pneumophila serogroup 1 clinical strains isolated in Italy. Int. J. Med. Microbiol..

[32] Hori J.I., Zamboni D.S. (2013). The mouse as a model for pulmonary legionella infection. Methods Mol. Biol..

[33] Rayamajhi M., Delgado C., Condon T., Riches D., Lenz L. (2012). Lung B cells promote early pathogen dissemination and hasten death from inhalation anthrax. Mucosal Immunol..

[34] Paudel S., Baral P., Ghimire L., Bergeron S., Jin L., DeCorte J.A., Le J.T., Cai S., Jeyaseelan S. (2019). CXCL1 regulates neutrophil homeostasis in pneumonia-derived sepsis caused by Streptococcus pneumoniae serotype 3. Blood.

[35] Osadnik T., Bujak K., Osadnik K., Czarnecka H., Pawlas N., Reguła R., Fronczek M., Lejawa M., Gawlita M., Gonera M. (2019). Novel inflammatory biomarkers may reflect subclinical inflammation in young healthy adults with obesity. Endokrynol. Pol..

[36] Calder P.C., Ahluwalia N., Albers R., Bosco N., Bourdet-Sicard R., Haller D., Holgate S.T., Jönsson L.S., Latulippe M.E., Marcos A. (2013). A Consideration of Biomarkers to be Used for Evaluation of Inflammation in Human Nutritional Studies. Br. J. Nutr..

[37] Hickman D.L. (2017). Evaluation of the neutrophil:lymphocyte ratio as an indicator of chronic distress in the laboratory mouse. Lab. Anim. (NY).

[38] Swan M.P., Hickman D.L. (2014). Evaluation of the neutrophil-lymphocyte ratio as a measure of distress in rats. Lab. Anim. (NY).

[39] Afriandi H.Y., Gatot P.A. (2020). Correlation of neutrophils lymphocytes ratio with femur muscle damage due to acute limb ischemia in white Wistar rats. Bali. Med. J..

[40] Dolma T., Mukherjee R., Mukherjee R., Pati B.K., De U.K. (2014). Acute Phase Response and Neutrophils: Lymphocyte Ratio in Response to Astaxanthin in Staphylococcal Mice Mastitis Model. J. Vet. Med..

[41] Marx G. (2003). Fluid therapy in sepsis with capillary leakage. Eur. J. Anaesthesiol..

[42] O’Connell K.E., Mikkola A.M., Stepanek A.M., Vernet A., Hall C.D., Sun C.C., Yildirim E., Staropoli J.F., Lee J.T., Brown D.E. (2015). Practical murine hematopathology: A comparative review and implications for research. Comp. Med..

[43] Qiu J., Xiaodi N., Wang J., Xing Y., Leng B., Dong J., Li H., Luo M., Zhang Y., Dai X. (2012). Capsaicin Protects Mice from Community-Associated Methicillin-Resistant Staphylococcus aureus Pneumonia. PLoS ONE.

[44] Bonaventura G., Pompilio A., Zappacosta R., Petrucci F., Fiscarelli E., Rossi C., Piccolomini R. (2010). Role of Excessive Inflammatory Response to Stenotrophomonas maltophilia Lung Infection in DBA/2 Mice and Implications for Cystic Fibrosis. Infect. Imunn..

[45] Fukushi M., Ito T., Oka T., Kitazawa T., Miyoshi-Akiyama T., Kirikae T., Yamashita M., Kudo K. (2011). Serial Histopathological Examination of the Lungs of Mice Infected with Influenza A Virus PR8 Strain. PLoS ONE.

[46] Fukushi M., Yamashita M., Miyoshi-Akiyama T., Kubo S., Yamamoto K., Kudo K. (2012). Laninamivir Octanoate and Artificial Surfactant Combination Therapy Significantly Increases Survival of Mice Infected with Lethal Influenza H1N1 Virus. PLoS ONE.

[47] Aeffner F., Bolon B., Davis I.C. (2015). Mouse Models of Acute Respiratory Distress Syndrome: A Review of Analytical Approaches, Pathologic Features, and Common Measurements. Toxicol. Pathol..

[48] Sordi R., Menezes-de-Lima O., Della-Justina A.M., Rezende E., Assreuy J. (2013). Pneumonia-induced sepsis in mice: Temporal study of inflammatory and cardiovascular parameters. Int. J. Exp. Pathol..

[49] Chastre J., Raghu G., Soler P., Brun P., Basset F., Gibert C. (1987). Pulmonary Fibrosis following Pneumonia Due to Acute Legionnaires’ Disease: Clinical, Ultrastructural, and Immunofluorescent Study. Chest.

[50] Carrington C.B. (1979). Pathology of Legionnaires’ disease. Ann. Intern. Med..

