# Supplementary material for: Acute Pneumonia Caused by Clinically Isolated Legionella pneumophila Sg 1, ST 62: Host Responses and Pathologies in Mice
Source: Microorganisms. 2022 Jan 14;10(1):179. doi: 10.3390/microorganisms10010179 (PMC8781576; doi:10.3390/microorganisms10010179)
Supplement: Supplementary file 1 [file microorganisms-10-00179-s001.zip › microorganisms-1531633-supplementary (1).pdf]

microorganisms

Supplementary materials

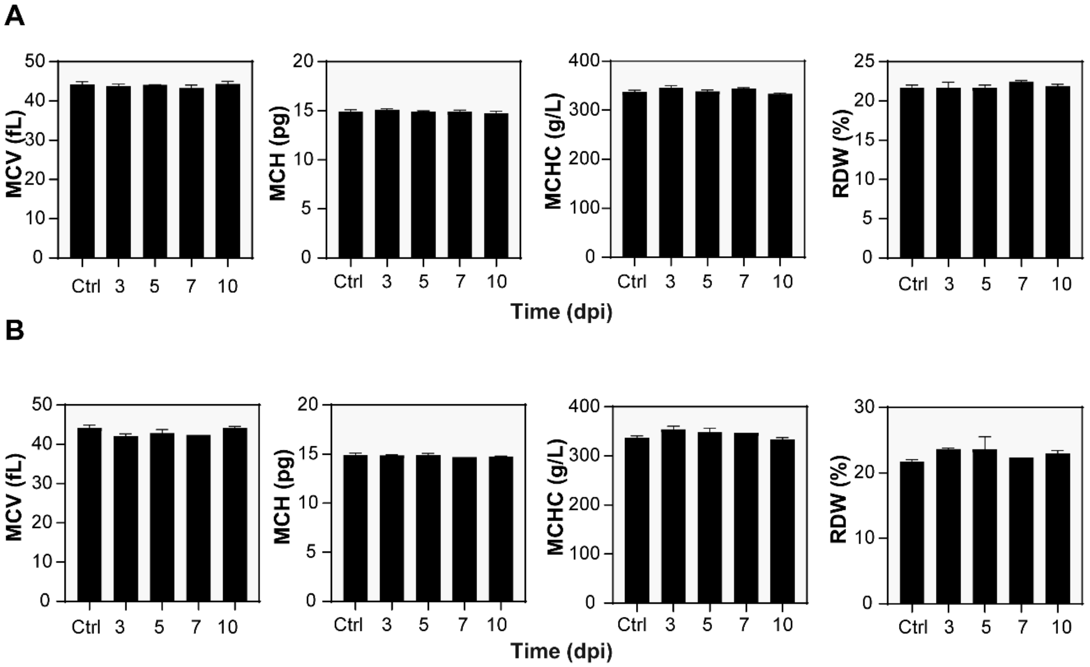

Figure S1. Hematological analysis parameters. Mice inoculated either with a low (**A**) or high (**B**) dose of *L. pneumophila* Sg 1, ST 62, and MAb Knoxville were anesthetized, and terminal blood collection was carried out via axilla incision. Data represent the mean ± SD. MCV—mean corpuscular volume; MCH—mean cell hemoglobin; MCHC—mean corpuscular hemoglobin concentration; RDW—red blood cell distribution width.

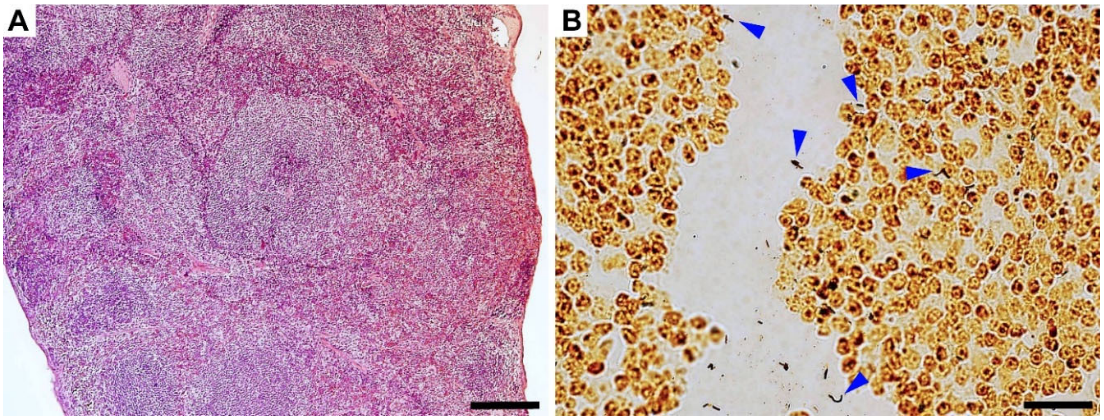

Figure S2. Disseminated *L. pneumophila* infection. Spleen histopathology (7 dpi, high-dose challenge) was assessed via (**A**) H&E staining and (**A**) Warthin–Starry staining. Rod-shaped structures, presumably *L. pneumophila*, were revealed (blue arrowheads). Scale bars: 200 and 20 μm, respectively.
